# Lower cerebral blood flow but not cerebrovascular response in elastin haploinsufficient mice

**DOI:** 10.1113/EP093234

**Published:** 2026-01-27

**Authors:** Abigail E. Cullen, Emily H. Reeve, Nick R. Winder, Grant D. Henson, Nayantara Arora, Thomas Leonhardt, Ainsley Hogan, Sahana Krishna Kumaran, Naly Setthavonsack, Victoria Krajbich, Art Riddle, Benjamin Zimmerman, Nabil J. Alkayed, Martin M. Pike, Randall L. Woltjer, Ashley E. Walker

**Affiliations:** ^1^ Human Physiology University of Oregon Eugene Oregon USA; ^2^ Pathology and Laboratory Medicine, School of Medicine Oregon Health and Science University Portland Oregon USA; ^3^ Knight Cardiovascular Institute Oregon Health and Science University Portland Oregon USA; ^4^ Biomedical Engineering, Advanced Imaging Research Center Oregon Health and Science University Portland Oregon USA; ^5^ Department of Pediatrics Oregon Health and Science University Portland Oregon USA; ^6^ Helfgott Research Institute National University of Natural Medicine Portland Oregon USA

**Keywords:** Brain volume, cerebrovascular reactivity, elastin haploinsufficiency, neuroinflammation

## Abstract

Elastin insufficiency is associated with structural differences in the large elastic arteries and cerebral artery dysfunction. However, previous studies have not assessed potential sex differences in cerebrovascular function. We measured cerebral blood flow (CBF) using arterial spin labeling MRI at rest and in response to hypercapnia challenge (cerebrovascular responsiveness, CR) in middle‐aged and old elastin haploinsufficient (*Eln^+/−^*) and wild‐type (*Eln^+/+^*) mice. We also assessed neuroinflammation by microglia and astrocyte cell counts. We found that *Eln^+/−^* mice had a significantly lower resting CBF in the cerebral cortex compared with *Eln^+/+^* mice, with similar non‐significant trends in the hippocampus and thalamus. In contrast, the *Eln^+/−^* mice had an intact hypercapnic response, resulting in better CR compared with *Eln^+/+^* in hippocampus, with a similar trend in the cerebral cortex. Sex did not impact CBF or CR. We found that *Eln^+/−^* mice had lower hippocampal volume compared with *Eln^+/+^* mice. Glia cell counts were highly dependent on brain region, with *Eln^+/−^* mice having more microglia in the cerebral cortex, but fewer astrocytes in the hippocampus compared with *Eln^+/+^* mice. While sex also impacted glial cell counts, we found no interactions between sex and *Eln* genotype. Our results demonstrate that elastin haploinsufficiency results in lower resting CBF, but greater CR.

## Abstract (highlights)

- **What is the central question of this study?**
  Do sex and elastin haploinsufficiency interact to impact cerebrovascular function and neuroinflammation?
- **What is the main finding and its importance?**
  Elastin haploinsufficiency led to lower resting cerebral blood flow and brain volumes and had region‐dependent effects on neuroinflammation. In contrast, elastin haploinsufficiency was associated with greater cerebrovascular responsiveness. Sex and elastin haploinsufficiency, however, did not interact to further impair these outcomes. These results suggest that structural changes in arterial elastin are not sufficient to independently impair cerebrovascular responsiveness.

## 1 INTRODUCTION

Elastin is a primary component of the vascular extracellular matrix that contributes to the elasticity of arteries and impacts cerebrovascular function (Reeve et al., 2024). Elastin haploinsufficient mice (*Eln^+/−^*) have stiffer arteries, at least for the large elastic arteries, compared with wild‐type mice (Li et al., 1998; Walker et al., 2015). These differences in artery structure coincide with impairments to cerebrovascular function. Our previous work demonstrates that *Eln^+/−^* mice have impaired cerebral artery endothelium‐dependent vasodilation and a greater vasoconstrictor responsiveness to angiotensin II (Walker et al., 2015, 2019). *Eln^+/−^* mice also have a lower cerebral blood flow (CBF) in the cerebral cortex than wild‐type mice (Knutsen et al., 2018). However, resting CBF provides limited information about cerebrovascular health, while cerebrovascular responsiveness (CR), such as the response to a hypercapnic stimulus, is more indicative of dynamic cerebrovascular function. Moreover, resting CBF and CR do not always follow the same trends. For example, in human subjects, greater aortic stiffness is associated with lower basal CBF, but higher CR (Jefferson et al., 2018). Thus, measuring CR provides a better understanding of the effects of elastin haploinsufficiency on cerebrovascular health.

There are significant sex differences in the trajectories of changes in arterial stiffness and cerebrovascular function with advancing age. The rate of increase in carotid artery stiffness is faster with ageing in females compared with age‐matched males (Lefferts et al., 2023). In addition, the age‐related declines in CBF and CR are greater in females than males (Aanerud et al., 2017; Kastrup et al., 1999). Sex differences are also found in the large arteries of aged *Eln^+/−^* mice, with the trajectories for changes in structural properties being different between male and female *Eln^+/−^* mice (Hawes et al., 2020). However, sex differences in cerebrovascular function in *Eln^+/−^* mice have not been previously studied.

Neuroinflammation and cerebrovascular dysfunction can amplify each other's dysfunction, leading to a vicious cycle that triggers cerebral atrophy. For example, reduced CBF can lead to hypoxia‐induced neuroinflammation (Tian et al., 2022). At the same time, chronically high levels of neuroinflammation contribute to blood–brain barrier disruption and vascular inflammation (Adamu et al., 2024). Neuroinflammation is characterized by an increase in glial cell activation, notably astrocytes and microglia (Adamu et al., 2024). There are sex‐specific differences in neuroinflammation, specifically, microglia isolated from female brains are more pro‐inflammatory than those from male brains (Coales et al., 2022), and the microglia from female brains are more strongly related to Alzheimer's disease pathogenesis (Casaletto et al., 2022). Cerebral atrophy is associated with both lower CBF and greater arterial stiffness, and potentially mediates the association of arterial stiffness and cognitive impairment (Brundel et al., 2012; Emrani et al., 2025; Li et al., 2024). Yet, the impact of elastin haploinsufficiency on neuroinflammation and brain volumes has not been previously studied.

In these studies, we set out to test the hypothesis that sex and elastin haploinsufficiency interact to impact cerebrovascular function and neuroinflammation, such that female *Eln^+/−^* mice would have worse CR and neuroinflammatory markers compared with male *Eln^+/−^* mice and *Eln^+/^* ***^+^*** mice of either sex. Our results do not support our hypothesis, as we found no interaction between *Eln* genotype and sex. However, we present several novel findings about the effects of elastin haploinsufficiency on cerebrovascular function and neuroinflammation.

## 2 METHODS

### 2.1 Ethical approval

All animal procedures conformed to the *Guide to the Care and Use of Laboratory Animals* and were approved by the Institutional Animal Care and Use Committee at the University of Oregon (AUP20‐25) and Oregon Health and Science University (TR03_IP00000147). Veterinarians at both University of Oregon and Oregon Health and Science University were consulted if any animals showed sign of decreased body weight, injury or illness. If any animals lost more than 20% of their starting body weight or were significantly injured or sick, they were removed from the study.

### 2.2 Animals

*Eln^+/−^* mice transferred from the University of Utah and the National Institutes of Health (Knutsen et al., 2018) were rederived with pathogen‐free C57BL/6J at the University of Oregon. Two months before magnetic resonance imaging (MRI), mice were transferred to Oregon Health and Science University. We studied males and females at ages of 14 to 22 months. The age range was due to research delays associated with the COVID‐19 pandemic. However, there were no differences in mean age between groups (M *Eln^+/+^*: 16.2 months, F *Eln^+/+^*: 16.2 months, M *Eln^+/−^*: 16.6 months, F *Eln^+/−^*: 16.3 months, all *P *> 0.05). After the MRI, mice were sacrificed by cardiac puncture and perfused with saline. Following saline, 4% paraformaldehyde was perfused to preserve the brain for histology. All mice were housed in an animal care facility on a 12/12 h light–dark cycle at 24°C with ad libitum access to standard chow (Lab Diet, Picolab Rodent Diet 20–5053, University of Oregon; Lab Diet, Laboratory Rodent Diet‐5001, Oregon Health and Science University) and water.

### 2.3 Arterial spin labelled MRI

MRI was performed at the OHSU Advanced Imaging Research Center using a Bruker‐Biospin 11.75 T small animal MR system with a ParaVision 6.0 software platform, 10 cm inner diameter gradient set with a 72 mm (ID) and 60 mm (length) radio frequency (RF) resonator for transmitting and an actively decoupled mouse head surface coil for receiving (Bruker‐Biospin, Billerica, MA, USA). Mice were anaesthetized with a ketamine/xylazine mixture (1.0 mg xylazine/7 mg ketamine/100 g) in combination with low isoflurane (0.75%) in 100% oxygen. The mice were positioned with heads immobilized on an animal cradle. Body temperature of the mice was monitored and maintained at 37°C while monitoring respiration (SA Instruments, Stony Brook, NY, USA). For each mouse, a coronal 25‐slice T2‐weighted image was acquired (ParaVision spin echo rapid acquisition with refocused echoes (RARE), 256 × 256 matrix, 125 µm in‐plane resolution, 0.5 mm slice width, repetition time (TR) 4000 ms, time to echo (TE) effective 23.64 ms, RARE factor 8, 2 averages). These T2‐weighted anatomical scans were used for positioning the blood flow image slice at a consistent position approximately 1.75 mm anterior to the anterior commissure. CBF (ml/min/100 g) was measured using arterial spin labelling (ASL), employing the flow‐sensitive alternating inversion recovery rapid acquisition with relaxation enhancement pulse sequence (ParaVision FAIR‐RARE), with TE/TR = 45.2/10000 ms, slice thickness = 1 mm, number of slices = 1, matrix = 128 × 128, 250 µm in‐plane resolution RARE factor = 72, and 23 turbo inversion recovery values ranging from 40 to 4400 ms, and acquisition time of 15 min. This sequence labels the inflowing blood by global inversion of the equilibrium magnetization (Kim, 1995). The ASL sequence was implemented first at the baseline condition with 100% oxygen, and subsequently 10 min after switching to the hypercapnia condition with 95%/5% oxygen/carbon dioxide. CBF maps (ml/100 g‐min) were generated using the Bruker Paravision ASL perfusion processing macro and exported into JIM 9 software (Xinapse Systems Ltd, West Bergholt, UK) for further processing. Outlier value brain pixels (outside 2 SDs) representing large arteries with high, pulsatile flow were excluded, thus arriving at flows which consistently represent tissue microvascular blood flow. The identical field of view geometry offsets of the T2 and ASL images enabled the regions of interest drawn on the T2 image to be readily overlaid onto the corresponding blood flow map for quantification. Mean blood flow was quantified for the whole brain, cortex, hippocampus and thalamus, each defined anatomically using the corresponding T2 images. ASL map voxels containing ventricular contributions were excluded. The Allen Brain Atlas as a guide to identify ventricles, hippocampus, cortex and thalamus. CR was calculated as the percentage increase in CBF from resting to hypercapnia.

### 2.4 Physiological monitoring

Continuous heart rate (HR) and respiratory rate (RR) were recorded using a Model 1025 monitoring system with respiration module (SA Instruments Inc., Stony Brook, NY, USA) during baseline and hypercapnic ASL measurements. HR and RR data were acquired at 1‐s intervals. Periods of poor signal quality due to animal movement or probe shifting and sudden change in gain factors in HR or RR were identified, and these data were removed. Diary plots for each HR and RR were generated by binning and averaging data over 30‐s intervals. If a bin lacked 10 s of data, it was omitted.

### 2.5 MRI anatomical analysis

We implemented a custom preprocessing pipeline using FSL (v6.0) (Jenkinson et al., 2012) and ANTs (v2.4.4) (Avants et al., 2011) to analyse mouse brain anatomy from high‐resolution T2‐weighted MRI data. T2‐weighted volumes were first bias‐field corrected using *N4BiasFieldCorrection* (Tustison et al., 2010) to mitigate intensity inhomogeneities. Brain extraction was performed using *bet4animal*. Each subject's brain‐extracted image was registered to a mouse anatomical reference atlas (Badhwar et al., 2013). Registration was carried out using *antsRegistration* (ANTs v2.4.4) with a multistage pipeline consisting of rigid, affine and SyN nonlinear transformations (Avants et al., 2011). Each step used mutual information and cross‐correlation metrics with multi‐resolution smoothing and shrink schedules to optimize spatial alignment. The resulting transformations were applied to the Badhwar atlas label map using *antsApplyTransforms* (Tustison et al., 2021). Quality control images and Jacobian determinant maps were generated for all registrations to verify transformation accuracy and assess local volumetric distortions. Regional masks were derived from the transformed atlas label map to quantify volumetric measures across key anatomical structures, including the hippocampus, cortex, thalamus, hypothalamus, amygdala, striatum, corpus callosum and ventricular system. Bilateral regions were merged to form composite masks where appropriate. Ventricular masks (including lateral, third and fourth ventricles) were combined and subtracted from the whole‐brain mask to derive a parenchymal (non‐ventricular) brain volume. Regional and whole‐brain volumes were quantified using *fslstats*, with voxel counts multiplied by voxel size to yield volume in mm^3^. All extracted measures were summarized in CSV format for statistical analysis.

### 2.6 Immunohistochemistry

After paraformaldehyde perfusion, brains were incubated in 4% paraformaldehyde for 48 h before being embedded in paraffin. Paraffinized brains were cut at 5 µm thickness and incubated with primary antibodies for Iba1 (ProteinTech, Rosemont, IL, USA, 10904‐1‐AP, 1:5000) or glial fibrillary acidic protein (GFAP) (ProteinTech, 16852‐1‐AP, 1:500), followed by development using Vector ELITE ABC kits (Vector Laboratories, Newark, CA, USA, PK‐6102). Visualization was done with 3,3′‐diaminobenzidine (Woltjer et al., 2015). Regions of interest (hippocampus, entorhinal cortex and thalamus) were determined using the Allen Brain Atlas and images were collected at a consistent area size (600 × 450 µm) using a Zeiss Axio Imager AZ10 (Zeiss Microscopy, Oberkochen, Germany) and analysed using ImageJ software (NIH, Bethesda, MD, USA) by percentage positive area and particle (cell) size.

### 2.7 Statistical analyses

Statistical analyses were performed with GraphPad Prism 10.2.3 (GraphPad Software, Boston, MA, USA). Two‐way analysis of variance (ANOVA) was used to determine interactions between elastin genotype and sex on CBF, CR and immunohistochemistry. GraphPad Prism 10.2.3 was used to calculate simple linear regression correlations between CBF, CR and neuroinflammation data. Significance was set at *P* < 0.05 and all values shown are means ± standard deviation. Outliers were determined to be values with *Z*‐scores outside the range of −2.0 to 2.0 and were removed from the data set. In cases of a significant *F*‐value, *post hoc* analyses were performed using the Tukey correction for preplanned comparisons. HR data during each ASL period were fit with a straight line using a least‐squares fit, and the line slopes were compared using the extra sum‐of‐squares *F*‐test. If data did not fit a normal distribution, we used transformation by log(*y*) or square root(*y*) to create normal distributions and proceeded with ANOVA and *post hoc* analysis.

## 3 RESULTS

### 3.1 Elastin haploinsufficiency has a stronger impact on resting CBF than sex

**FIGURE 1:**
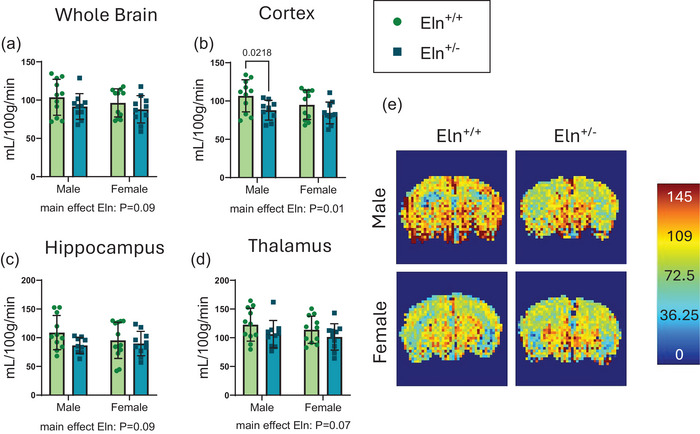
Elastin haploinsufficiency is associated with lower resting CBF. (a–d) For male and female elastin wild‐type (*Eln^+/+^*, *n* = 11 M, *n* = 10–13 F) and haploinsufficient (*Eln^+/−^*, *n* = 9–10 M, *n* = 10 F) aged mice (16.2 ± 2.6 months), blood flow of the whole brain, cortex, hippocampus and thalamus was measured via arterial spin labeling MRI in resting conditions. (e) Representative images. All data are shown as means ± SD. A 2‐way ANOVA result *P *< 0.05 was considered significant.

**FIGURE 2:**
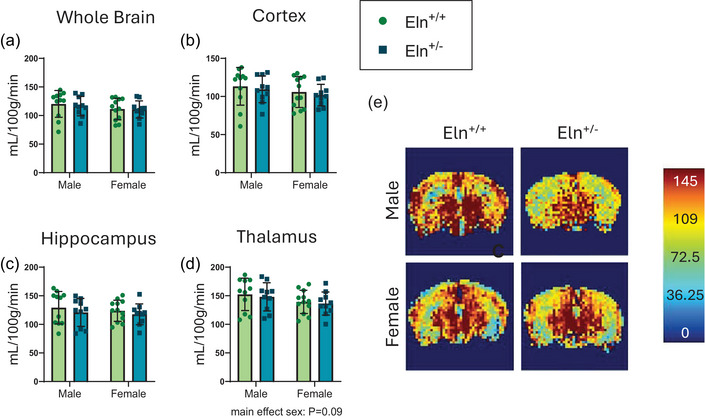
Elastin haploinsufficiency does not impact CBF in hypercapnic conditions. (a–d) For male and female elastin wild‐type (*Eln^+/+^*, *n* = 11 M, *n* = 12 F) and haploinsufficient (*Eln^+/−^*, *n* = 10 M, *n* = 10 F) aged mice (16.2 ± 2.6 months), blood flow of the whole brain, cortex, hippocampus and thalamus was measured via arterial spin labelling MRI in hypercapnic conditions. (e) Representative images. All data are shown as means ± SD. A 2‐way ANOVA result *P *< 0.05 was considered significant.

Resting CBF in the cortex was significantly lower in *Eln^+/−^* mice compared with *Eln^+/^* ***^+^*** mice (Figure 1b, *P* = 0.01). *Post hoc* analysis indicated a significant difference between the male *Eln^+/^* ***^+^*** and *Eln^+/−^* mice (Figure 1b, *P* = 0.02). We found non‐significant trends for main effects of elastin haploinsufficiency in the whole brain, hippocampus and thalamus (Figure 1a,c,d, all *P *< 0.10). In contrast, sex had no effect on resting CBF of any brain region (Figure 1a–d, all *P *> 0.05). During hypercapnia, there were no effects of sex or elastin haploinsufficiency on CBF of any brain region (Figure 2a–d, all *P *> 0.05).

**FIGURE 3:**
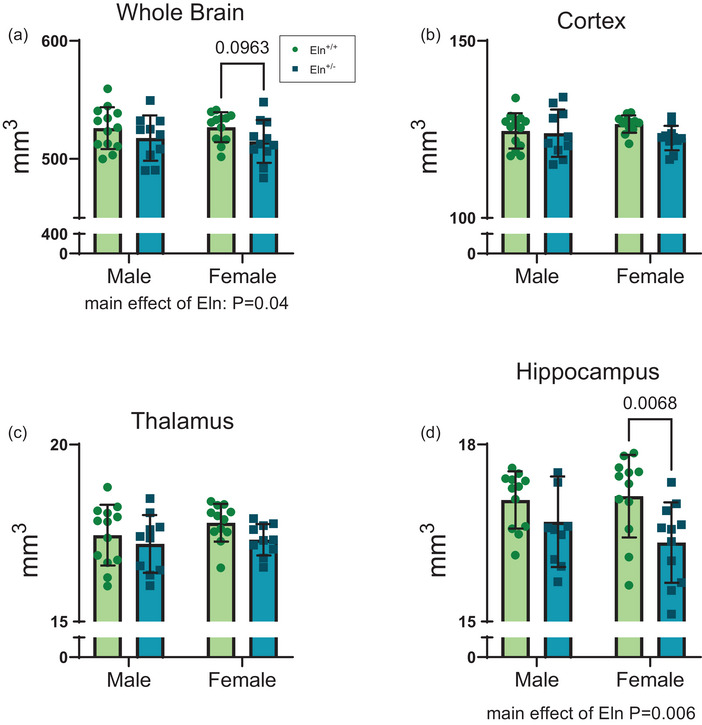
Elastin haploinsufficiency impacts regional brain volumes. Volume (mm^3^) was determined for the whole brain (a), cortex (b), thalamus (c), and hippocampus (d) in elastin wild‐type (*Eln^+/+^*, *n* = 12‐13 M, *n* = 13 F) and haploinsufficient mice (*Eln^+/−^*, *n* = 10 M, *n* = 10–11 F). A main effect of elastin haploinsufficiency was found in the whole brain and hippocampus (*P* = 0.04 and 0.006, respectively). Between female mice, there was a significant effect of elastin haploinsufficiency in the hippocampus (*P* = 0.007) and a trend for this in the whole brain (*P* = 0.09). All data are shown as means ± SD. A 2‐way ANOVA result *P *< 0.05 was considered significant.

We also measured the volume of the whole brain and various regions of interest and identified a significant effect of elastin haploinsufficiency. Overall, there was significantly less volume for the whole brain in *Eln^+/−^* mice compared with *Eln^+/^* ***^+^*** mice (Figure 3a, *P* = 0.04). There was also a main effect of elastin haploinsufficiency on hippocampal volume where the *Eln^+/−^* mice had significantly less volume compared to *Eln^+/^* ***^+^*** mice (Figure 3d, *P* = 0.006). While we found these differences, other regions of interest (cortex and thalamus) were not impacted significantly by genotype or sex (Figure 3b,c, both *P *> 0.05). We also analysed volumes for other brain regions, including the amygdala, corpus callosum, striatum and the ventricles and observed no significant differences or any region due to genotype or sex (Supporting information, Table S1, all *P *> 0.05).

### 3.2 Elastin haploinsufficiency has a stronger impact on CR than sex

**FIGURE 4:**
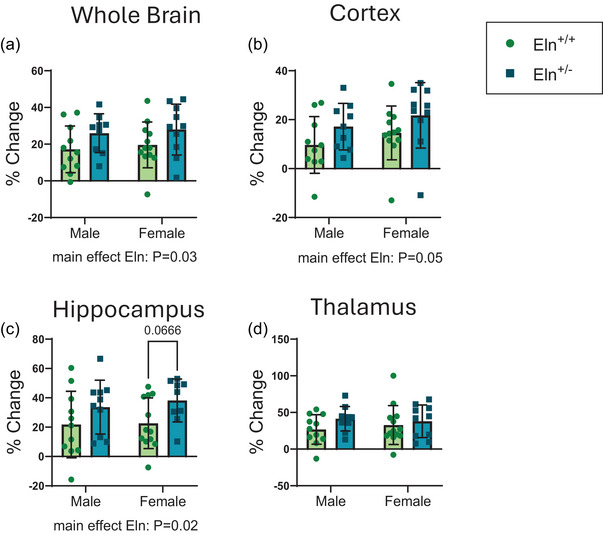
Elastin haploinsufficiency is associated with greater CR. For male and female elastin wild‐type (*Eln^+/+^*, *n* = 10–11 M, *n* = 12–13 F) and haploinsufficient (*Eln^+/−^*, *n* = 10 M, *n* = 10 F) aged mice (16.2 ± 2.6 months), cerebrovascular responsiveness (CR) was calculated between resting and hypercapnic blood flow measured via arterial spin labelling MRI (a–d). All data are shown as means ± SD. A 2‐way ANOVA result *P *< 0.05 was considered significant.

We then calculated CR as the percentage change in CBF between resting and hypercapnic CBF. We found a main effect of elastin haploinsufficiency on CR in the whole brain and hippocampus (Figure 4a,c, *P *< 0.05) and a trend in the cortex (Figure 4b, *P* = 0.05), such that the *Eln^+/−^* mice had a greater CR compared with the *Eln^+/^* ***^+^*** mice. We found no effect of sex on CR in any brain region (Figure 4a–d, *P *> 0.05).

We monitored RR and HR before and during baseline and hypercapnia ASL scans in a subset of animals (Supporting information, Figure S1). No differences in baseline or hypercapnia RR were observed between genotypes. A small non‐significant decrease in RR was observed during hypercapnia in *Eln^+/^* ***^+^*** mice (Supporting information, Figure S1A, *P* = 0.01). The diary plot for HR showed a consistent increase in both genotypes across the monitoring period (Supporting information, Figure S1B). Therefore, the slopes of the HR increase were compared between conditions and genotypes and found to be equivalent (*P *> 0.05).

### 3.3 Sex and elastin haploinsufficiency impact markers of neuroinflammation

**FIGURE 5:**
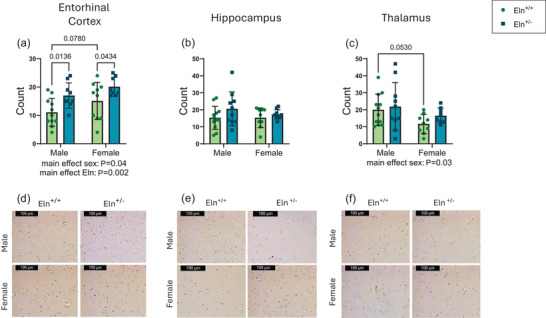
Sex has a greater influence over microglia than elastin haploinsufficiency. (a–c) Iba1‐positive cell count in the entorhinal cortex, hippocampus and thalamus, respectively, in male and female elastin wild‐type (*Eln^+/+^*, *n* = 11–12 M, *n* = 7–10 F) and haploinsufficient (*Eln^+/−^*, *n* = 9–10 M, *n* = 8–9 F) mice. (g–i) Representative images. All data are shown as means ± SD. A 2‐way ANOVA result *P *< 0.05 was considered significant.

**FIGURE 6:**
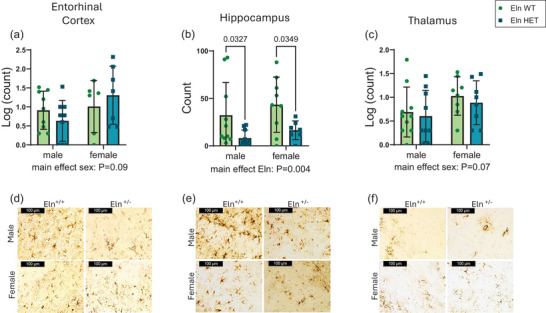
Sex and elastin haploinsufficiency influence astrocytes. (a–c) GFAP‐positive cell count for the entorhinal cortex (a), hippocampus (b), and thalamus (c) for elastin wild‐type (*Eln^+/+^*, *n* = 10–12 M, *n* = 8–9 F) and haploinsufficient (*Eln^+/−^*, *n* = 9–10 M, *n* = 7–9 F). (d–f) Representative images. Non‐Gaussian data were normalized via log(count). All data are shown as means ± SD. A 2‐way ANOVA result *P *< 0.05 was considered significant.

We assessed markers of neuroinflammation by measuring Iba1‐positive cells, a marker of microglia, and GFAP‐positive cells, a marker of activated astrocytes. For Iba1, there was a strong main effect of elastin haploinsufficiency within the entorhinal cortex, indicating more Iba1‐positive cells in *Eln^+/−^* mice compared with *Eln^+/^* ***^+^*** mice (Figure 5a, *P* = 0.002), but this was not significantly different in the hippocampus or thalamus (Figure 5b,c, *P *> 0.05). We also found a main effect of sex on Iba1‐positive cells in the entorhinal cortex and thalamus regions (Figure 5a,c, *P *< 0.05), such that females had a higher cell count in the entorhinal cortex while males had a higher cell count in the thalamus. *Post hoc* analysis of the entorhinal cortex found significant differences in Iba1 cell count between male *Eln^+/^* ***^+^*** and *Eln^+/−^* mice, female *Eln^+/^* ***^+^*** and *Eln^+/−^* mice, and between male and female *Eln^+/^* ***^+^*** mice. *Post hoc* analysis of the thalamus data indicated significantly fewer Iba1‐positive cells between male *Eln^+/^* ***^+^*** and female *Eln^+/^* ***^+^*** mice. For GFAP, we found a main effect of genotype on cell count in the hippocampus (Figure 6b, *P* = 0.004), such that there were fewer GFAP‐positive cells in the *Eln^+/−^* mice compared with *Eln^+/^* ***^+^*** mice. However, there was no effect of genotype on GFAP‐positive cell count in the entorhinal cortex or thalamus (Figure 6a,c, *P *> 0.05). There were no significant main effects of sex or interactions with sex for GFAP in any brain region (Figure 5a‐c, *P *> 0.05). We analysed associations between haemodynamic measures and neuroinflammation outcomes, and found no significant correlations (all *P *> 0.05).

## 4 DISCUSSION

In this study, we examined the impact of elastin haploinsufficiency on resting CBF, CR and neuroinflammation, and further explored the interaction with sex for these outcomes. We found that although elastin haploinsufficiency leads to lower resting CBF in the cerebral cortex, it is associated with intact hypercapnic response and better CR. We also found that elastin haploinsufficiency leads to lower volumes for the whole brain and hippocampus. We found that neuroinflammation was highly dependent on brain region, and in general, female sex was associated with a greater number of microglia. Elastin haploinsufficiency was associated with more microglia but fewer astrocytes. In summary, we find that elastin haploinsufficiency has disparate effects on resting CBF and CR as well as brain region‐dependent effects on neuroinflammation, but there is no interaction of elastin haploinsufficiency and sex on these outcomes.

Our data, together with previous studies, indicate that elastin haploinsufficiency is associated with lower cortical blood flow throughout the lifespan. Our findings in middle‐aged and old mice align with a previous study by Knutsen et al. in young (4 months) *Eln^+/−^* mice. They found that resting CBF was lower in *Eln^+/−^* mice compared with *Eln^+/^* ***^+^*** mice (Knutsen et al., 2018). They also found a similar pattern for regional impacts, with the most significant effects of elastin haploinsufficiency in the cortex (Knutsen et al., 2018). Cortical atrophy is significantly related to cognitive decline in Alzheimer's disease (Mouton et al., 1998) and the entorhinal cortex is a crucial site of initiation for Alzheimer's pathology (Igarashi, 2023). Thus, this reduction in cortical blood flow with elastin haploinsufficiency could impact cognitive function and Alzheimer's disease onset. This is supported by our study where we also find that *Eln^+/−^* mice have lower brain volumes. However, while resting CBF has been associated with cognitive decline and disease (Korte et al., 2020), a more physiologically relevant measure of dynamic changes in CBF is CR. Interestingly, we found that elastin haploinsufficiency was associated with higher CR across multiple brain regions, likely attributed to lower baseline CBF and intact hypercapnic response. Our CR data agree with Jefferson et al., who found that higher aortic stiffness is associated with higher CR in cognitively normal older adults. Similar to our study, these correlations with CR were found across multiple brain regions (Jefferson et al., 2018). This greater CR in elastin haploinsufficient mice and older adults with stiffer large arteries could be mediated by the lower resting CBF, thus representing a ceiling effect in the wild‐type and lower stiffness groups. Therefore, it remains unclear whether the higher CR represents a beneficial or detrimental physiological response.

The CR to hypercapnia is dependent on baseline CBF and arterial $P_{CO_{2}}$. The typical homeostatic response to increased arterial $P_{CO_{2}}$ and reduced pH in awake animals is tachypnoea, which rapidly normalizes pH and reduces $P_{CO_{2}}$. We found no significant increase in RR in anaesthetized mice of either *Eln^+/+^* or *Eln^+/−^* genotype after hypercapnia exposure. Thus, we expect that the observed ASL signal changes likely reflect altered CBF under these conditions. Unexpectedly, we observed a non‐significant reduction in RR from baseline to hypercapnia conditions in *Eln^+/+^* mice. Potentially, the use of an inhaled anaesthetic could explain the unexpected response, but future studies are needed. As there was no change in RR in the *Eln^+/−^* group, this could be a confounding factor in the CR outcomes; however, we did not observe differences between genotypes for RR during hypercapnia. Importantly, HR was similar between genotypes for conditions, suggesting that the level of anaesthesia did not differ between groups.

By influencing neuroinflammation and neurovascular coupling, glia impact CBF. At the same time, impaired CBF can aggravate neuroinflammation. Microglia can play contradictory roles in the brain: what starts as a protective role by removing pathological protein aggregates can transition to a harmful role, promoting uncontrolled inflammation (Gao et al., 2023; Hansen et al., 2018; Miao et al., 2023). We found that there were significantly more microglia in the entorhinal cortex of *Eln^+/−^* mice compared with *Eln^+/^* ***^+^*** mice, which is consistent with greater inflammation leading to lower CBF. Astrocytes are primary controllers of neurovascular coupling (Stackhouse & Mishra, 2021) but can also become reactive and pro‐inflammatory (Kim et al., 2024). As such, our finding of fewer astrocytes in the *Eln^+/−^* mice is contradictory to our resting CBF and CR findings, particularly as it has been shown that astrocytes play a role in vasodilation in response to hypercapnia (Howarth et al., 2017). In summary, our data potentially suggest that the differences in microglia, rather than astrocytes, have a greater influence on CBF and CR with elastin haploinsufficiency.

Where we have identified similar effects of elastin haploinsufficiency for males and females, previous reports have indicated that only males are impacted. Hawes et al. (2020) examined sex differences in male and female *Eln^+/^* ***^+^*** and *Eln^+/−^* mice, and found that elastin haploinsufficiency impacted only male mice, specifically for pulse pressure, collagen and elastin. Similarly, Kailash et al. (2024) found that material stiffness was impacted in only male *Eln^+/−^* mice. Conversely, while we find sex differences in astrocyte and microglia counts, these did not interact with *Eln* genotype. Taken together, our findings and previous studies suggest that there are sex differences in the effects of elastin haploinsufficiency on large arteries but not the cerebral microvasculature.

Our study is not without limitations. The *Eln^+/−^* model is a whole‐body genetic manipulation, and thus we cannot determine if the effects are due to a direct impact of the genotype on the cerebral vasculature or if the cerebrovascular effects are indirectly caused by differences in large artery structure. We also did not assess the arterial partial pressure of CO_2_, and thus cannot discern if the differences in CR were due to differences in the CO_2_ stimulus between the mice. Nor did we measure sex hormones, which are known to impact cardiovascular health. Our method of anaesthesia, while common for rodent studies, is a limitation (Bah et al., 2022). Lei et al. (2001) identified that ketamine and xylazine in combination reduce forebrain CBF. We also did not measure blood pressure during the MRI, and as such, do not know if differences in perfusion pressure impact the measures of CBF. Future studies on CBF and CR would benefit from the inclusion of several key data points during primary data collection including but not limited to arterial partial pressure of CO_2_ and blood pressure.

Overall, our findings suggest that elastin haploinsufficiency leads to lower resting CBF and brain volume but greater CR, and sex differences do not influence these responses. These results may suggest that structural changes to arterial elastin are not sufficient to independently impair CR.

## AUTHOR CONTRIBUTIONS

Ashley E. Walker, Martin M. Pike and Nabil J. Alkayed conceived and designed research, Abigail E. Cullen, Emily H. Reeve, Nick R. Winder, Grant D. Henson, Nayantara Arora, Thomas Leonhardt, Ainsley Hogan, S.K., Naly Setthavonsack, Victoria Krajbich, Martin M. Pike and Randall L. Woltjer performed experiments, Abigail E. Cullen, Emily H. Reeve, Zimmerman, Art Riddle and Ashley E. Walker analysed data, Abigail E. Cullen, Emily H. Reeve and Ashley E. Walker interpreted results of experiments, Abigail E. Cullen prepared figures, Abigail E. Cullen and Ashley E. Walker drafted manuscript, Abigail E. Cullen, Emily H. Reeve, Nick R. Winder, Grant D. Henson, Nayantara Arora, Thomas Leonhardt, Ainsley Hogan, S.K., Naly Setthavonsack, Victoria Krajbich, Benjamin Zimmerman, Art Riddle, Martin M. Pike, Nabil J. Alkayed, Randall L. Woltjer and Ashley E. Walker edited and revised manuscript and approved final version of manuscript. All authors have read and approved the final version of this manuscript and agree to be accountable for all aspects of the work in ensuring that questions related to the accuracy or integrity of any part of the work are appropriately investigated and resolved. All persons designated as authors qualify for authorship, and all those who qualify for authorship are listed.

## CONFLICTS OF INTEREST

None declared.

## Supporting information



Figure S1. Physiological responses to CO_2_ exposure in anaesthetized *Eln^+/+^* and *Eln^+/−^* mice.
Table S1. Brain region volumes (mm^3^).

## Data Availability

Data will be made available upon reasonable request.

## References

[eph70191-bib-0001] Aanerud, J. , Borghammer, P. , Rodell, A. , Jonsdottir, K. Y. , & Gjedde, A. (2017). Sex differences of human cortical blood flow and energy metabolism. Journal of Cerebral Blood Flow and Metabolism, 37(7), 2433–2440. 27629099 10.1177/0271678X16668536 PMC5531342

[eph70191-bib-0002] Adamu, A. , Li, S. , Gao, F. , & Xue, G. (2024). The role of neuroinflammation in neurodegenerative diseases: Current understanding and future therapeutic targets. Frontiers in Aging Neuroscience, 16, 1347987. 38681666 10.3389/fnagi.2024.1347987 PMC11045904

[eph70191-bib-0003] Avants, B. B. , Tustison, N. J. , Song, G. , Cook, P. A. , Klein, A. , & Gee, J. C. (2011). A reproducible evaluation of ANTs similarity metric performance in brain image registration. Neuroimage, 54(3), 2033–2044. 20851191 10.1016/j.neuroimage.2010.09.025 PMC3065962

[eph70191-bib-0004] Badhwar, A. , Lerch, J. P. , Hamel, E. , & Sled, J. G. (2013). Impaired structural correlates of memory in Alzheimer's disease mice. Neuroimage Clinical, 3, 290–300. 24273714 10.1016/j.nicl.2013.08.017 PMC3814975

[eph70191-bib-0005] Bah, T. M. , Allen, E. M. , Garcia‐Jaramillo, M. , Perez, R. , Zarnegarnia, Y. , Davis, C. M. , Bloom, M. B. , Magana, A. A. , Choi, J. , Bobe, G. , Pike, M. M. , Raber, J. , Maier, C. S. , & Alkayed, N. J. (2022). GPR39 deficiency impairs memory and alters oxylipins and inflammatory cytokines without affecting cerebral blood flow in a high‐fat diet mouse model of cognitive impairment. Frontiers in Cellular Neuroscience, 16, 893030. 35875352 10.3389/fncel.2022.893030 PMC9298837

[eph70191-bib-0006] Brundel, M. , van den Berg, E. , Reijmer, Y. D. , de Bresser, J. , Kappelle, L. J. , Biessels, G. J. , & Utrecht Diabetic Encephalopathy Study group . (2012). Cerebral haemodynamics, cognition and brain volumes in patients with type 2 diabetes. Journal of Diabetes and Its Complications, 26(3), 205–209. 22520398 10.1016/j.jdiacomp.2012.03.021

[eph70191-bib-0007] Casaletto, K. B. , Nichols, E. , Aslanyan, V. , Simone, S. M. , Rabin, J. S. , & La Joie, R. (2022). Sex‐specific effects of microglial activation on Alzheimer's disease proteinopathy in older adults. Brain, 145(10), 3536–3545. 35869598 10.1093/brain/awac257 PMC10233295

[eph70191-bib-0008] Coales, I. , Tsartsalis, S. , Fancy, N. , Weinert, M. , Clode, D. , Owen, D. , & Matthews, P. M. (2022). Alzheimer's disease‐related transcriptional sex differences in myeloid cells. Journal of Neuroinflammation, 19(1), 247. 36199077 10.1186/s12974-022-02604-w PMC9535846

[eph70191-bib-0009] Emrani, S. , Tanley, J. , Schaich, C. L. , Shah, S. , Bertoni, A. G. , Korcarz, C. , Heckbert, S. R. , Habes, M. , Lockhart, S. N. , Chirinos, J. A. , Ding, J. , Stein, J. H. , Gepner, A. D. , Bryan, R. N. , Nasrallah, I. M. , Luchsinger, J. A. , Hayden, K. M. , Liu, Y. , & Hughes, T. M. (2025). Carotid and regional arterial stiffness and dementia‐related imaging biomarkers in the multi‐ethnic study of atherosclerosis (MESA). Alzheimers Dementia, 21(10), e70688. 10.1002/alz.70688 PMC12531903 41104604

[eph70191-bib-0010] Gao, C. , Jiang, J. , Tan, Y. , & Chen, S. (2023). Microglia in neurodegenerative diseases: Mechanism and potential therapeutic targets. Signal Transduction and Targeted Therapy, 8(1), 359. 37735487 10.1038/s41392-023-01588-0 PMC10514343

[eph70191-bib-0011] Hansen, D. V. , Hanson, J. E. , & Sheng, M. (2018). Microglia in Alzheimer's disease. Journal of Cell Biology, 217(2), 459–472. 29196460 10.1083/jcb.201709069 PMC5800817

[eph70191-bib-0012] Hawes, J. Z. , Cocciolone, A. J. , Cui, A. H. , Griffin, D. B. , Staiculescu, M. C. , Mecham, R. P. , & Wagenseil, J. E. (2020). Elastin haploinsufficiency in mice has divergent effects on arterial remodeling with aging depending on sex. American Journal of Physiology‐Heart and Circulatory Physiology, 319(6), H1398–H408. 33035438 10.1152/ajpheart.00517.2020 PMC7792709

[eph70191-bib-0013] Howarth, E. (2017). A critical role for astrocytes in hypercapnic vasodilation in brain. Journal of Neuroscience, 37(18), 4860. 28137973 10.1523/JNEUROSCI.0005-16.2016 PMC5354350

[eph70191-bib-0014] Igarashi, K. M. (2023). Entorhinal cortex dysfunction in Alzheimer's disease. Trends in Neuroscience, 46(2), 124–136. 10.1016/j.tins.2022.11.006 PMC9877178 36513524

[eph70191-bib-0015] Jefferson, A. L. , Cambronero, F. E. , Liu, D. , Moore, E. E. , Neal, J. E. , Terry, J. G. , Nair, S. , Pechman, K. R. , Rane, S. , Davis, L. T. , Gifford, K. A. , Hohman, T. J. , Bell, S. P. , Wang, T. J. , Beckman, J. A. , & Carr, J. J. (2018). Higher aortic stiffness is related to lower cerebral blood flow and preserved cerebrovascular reactivity in older adults. Circulation, 138(18), 1951–1962. 30018169 10.1161/CIRCULATIONAHA.118.032410 PMC6394409

[eph70191-bib-0016] Jenkinson, M. , Beckmann, C. F. , Behrens, T. E. , Woolrich, M. W. , & Smith, S. M. (2012). FSL. Neuroimage, 62(2), 782–790. 21979382 10.1016/j.neuroimage.2011.09.015

[eph70191-bib-0017] Kailash, K. A. , Hawes, J. Z. , Cocciolone, A. J. , Bersi, M. R. , Mecham, R. P. , & Wagenseil, J. E. (2024). Constitutive modeling of mouse arteries suggests changes in directional coupling and extracellular matrix remodeling that depend on artery type, age, sex, and elastin amounts. Journal of Biomechanical Engineering, 146(5), 060901. 37646627 10.1115/1.4063272 PMC12410933

[eph70191-bib-0018] Kastrup, A. , Happe, V. , Hartmann, C. , & Schabet, M. (1999). Gender‐related effects of indomethacin on cerebrovascular CO_2_ reactivity. Journal of the Neurological Sciences, 162(2), 127–132. 10202978 10.1016/s0022-510x(98)00288-3

[eph70191-bib-0019] Kim, J. , Yoo, I. D. , Lim, J. , & Moon, J. S. (2024). Pathological phenotypes of astrocytes in Alzheimer's disease. Experimental & Molecular Medicine, 56(1), 95–99. 38172603 10.1038/s12276-023-01148-0 PMC10834520

[eph70191-bib-0020] Kim, S. G. (1995). Quantification of relative cerebral blood flow change by flow‐sensitive alternating inversion recovery (FAIR) technique: Application to functional mapping. Magnetic Resonance in Medicine, 34(3), 293–301. 7500865 10.1002/mrm.1910340303

[eph70191-bib-0021] Knutsen, R. H. , Beeman, S. C. , Broekelmann, T. J. , Liu, D. , Tsang, K. M. , Kovacs, A. , Ye, L. , Danback, J. R. , Watson, A. , Wardlaw, A. , Wagenseil, J. E. , Garbow, J. R. , Shoykhet, M. , & Kozel, B. A. (2018). Minoxidil improves vascular compliance, restores cerebral blood flow, and alters extracellular matrix gene expression in a model of chronic vascular stiffness. American Journal of Physiology‐Heart and Circulatory Physiology, 315(1), H18–H32. 29498532 10.1152/ajpheart.00683.2017 PMC6087770

[eph70191-bib-0022] Korte, N. , Nortley, R. , & Attwell, D. (2020). Cerebral blood flow decrease as an early pathological mechanism in Alzheimer's disease. Acta Neuropathologica, 140(6), 793–810. 32865691 10.1007/s00401-020-02215-w PMC7666276

[eph70191-bib-0023] Lefferts, W. K. , Reed, K. S. , Rosonke, R. E. , Augustine, J. A. , & Moreau, K. L. (2023). Age‐associated increases in middle cerebral artery pulsatility differ between men and women. American Journal of Physiology‐Heart and Circulatory Physiology, 325(5), H1118–H1125. 37682233 10.1152/ajpheart.00453.2023 PMC10908402

[eph70191-bib-0024] Lei, H. , Grinberg, O. , Nwaigwe, C. I. , Hou, H. G. , Williams, H. , Swartz, H. M. , & Dunn, J. F. (2001). The effects of ketamine‐xylazine anesthesia on cerebral blood flow and oxygenation observed using nuclear magnetic resonance perfusion imaging and electron paramagnetic resonance oximetry. Brain Research, 913(2), 174–179. 11549383 10.1016/s0006-8993(01)02786-x

[eph70191-bib-0025] Li, D. Y. , Brooke, B. , Davis, E. C. , Mecham, R. P. , Sorensen, L. K. , & Boak, B. B. (1998). Elastin is an essential determinant of arterial morphogenesis. Nature, 393(6682), 276–280. 9607766 10.1038/30522

[eph70191-bib-0026] Li, X. , Xing, J. , Hui, Y. , Shi, H. , Li, R. , Zhang, S. , Chen, S. , Li, J. , Liang, X. , Wu, Y. , Zhao, P. , Wu, S. , & Wang, Z. (2024). Hippocampal volume mediates the association of arterial stiffness with cognitive impairment in adult population. Journal of Hypertension, 42(9), 1566–1572. 38747362 10.1097/HJH.0000000000003760 PMC11296271

[eph70191-bib-0027] Miao, J. , Ma, H. , Yang, Y. , Liao, Y. , Lin, C. , Zheng, J. , Yu, M. , & Lan, J. (2023). Microglia in Alzheimer's disease: Pathogenesis, mechanisms, and therapeutic potentials. Frontiers in aging neuroscience, 15, 1201982. 37396657 10.3389/fnagi.2023.1201982 PMC10309009

[eph70191-bib-0028] Mouton, P. R. , Martin, L. J. , Calhoun, M. E. , Dal Forno, G. , & Price, D. L (1998). Cognitive decline strongly correlates with cortical atrophy in Alzheimer's dementia. Neurobiology of Aging, 19(5), 371–377. 9880038 10.1016/s0197-4580(98)00080-3

[eph70191-bib-0029] Reeve, E. H. , Barnes, J. N. , Moir, M. E. , & Walker, A. E. (2024). Impact of arterial stiffness on cerebrovascular function: A review of evidence from humans and preclincal models. American Journal of Physiology‐Heart and Circulatory Physiology, 326(3), H689–H704. 38214904 10.1152/ajpheart.00592.2023 PMC11221809

[eph70191-bib-0030] Stackhouse, T. L. , & Mishra, A. (2021). Neurovascular coupling in development and disease: Focus on astrocytes. Frontiers in Cell and Developmental Biology, 9, 702832. 34327206 10.3389/fcell.2021.702832 PMC8313501

[eph70191-bib-0031] Tian, Z. , Ji, X. , & Liu, J. (2022). Neuroinflammation in vascular cognitive impairment and dementia: Current evidence, advances, and prospects. International Journal of Molecular Sciences, 23(11), 6224. 35682903 10.3390/ijms23116224 PMC9181710

[eph70191-bib-0032] Tustison, N. J. , Avants, B. B. , Cook, P. A. , Zheng, Y. , Egan, A. , & Yushkevich, P. A. (2010). N4ITK: Improved N3 bias correction. Institute of Electrical and Electronics Engineers Transactions on Medical Imaging, 29(6), 1310–1320. 10.1109/TMI.2010.2046908 PMC3071855 20378467

[eph70191-bib-0033] Tustison, N. J. , Cook, P. A. , Holbrook, A. J. , Johnson, H. J. , Muschelli, J. , Devenyi, G. A. , Duda, J. T. , Das, S. R. , Cullen, N. C. , Gillen, D. L. , Yassa, M. A. , Stone, J. R. , Gee, J. C. , & Avants, B. B. (2021). The ANTsX ecosystem for quantitative biological and medical imaging. Scientific Reports, 11(1), 9068. 33907199 10.1038/s41598-021-87564-6 PMC8079440

[eph70191-bib-0034] Walker, A. E. , Henson, G. D. , Reihl, K. D. , Morgan, R. G. , Dobson, P. S. , Nielson, E. I. , Ling, J. , Mecham, R. P. , Li, D. Y. , Lesniewski, L. A. , & Donato, A. J. (2015). Greater impairments in cerebral artery compared with skeletal muscle feed artery endothelial function in a mouse model of increased large artery stiffness. The Journal of Physiology, 593(8), 1931–1943. 25627876 10.1113/jphysiol.2014.285338 PMC4405752

[eph70191-bib-0035] Walker, A. E. , Kronquist, E. K. , Chinen, K. T. , Reihl, K. D. , Li, D. Y. , Lesniewski, L. A. , & Donato, A. J. (2019). Cerebral and skeletal muscle feed artery vasoconstrictor responses in a mouse model with greater large elastic artery stiffness. Experimental Physiology, 104(3), 434–442. 30633428 10.1113/EP087453 PMC7079737

[eph70191-bib-0036] Woltjer, R. L. , Reese, L. C. , Richardson, B. E. , Tran, H. , Green, S. , Pham, T. , Chalupsky, M. , Gabriel, I. , Light, T. , Sanford, L. , Jeong, S. Y. , Hamada, J. , Schwanemann, L. K. , Rogers, C. , Gregory, A. , Hogarth, P. , & Hayflick, S. J. (2015). Pallidal neuronal apolipoprotein E in pantothenate kinase‐associated neurodegeneration recapitulates ischemic injury to the globus pallidus. Molecular Genetics and Metabolism, 116(4), 289–297. 26547561 10.1016/j.ymgme.2015.10.012 PMC4688119

