# Supplementary material for: Lower cerebral blood flow but not cerebrovascular response in elastin haploinsufficient mice
Source: Exp Physiol. 2026 Jan 27:10.1113/EP093234. Online ahead of print. doi: 10.1113/EP093234 (PMC13394619; doi:10.1113/EP093234)
Supplement: Supplementary file 1 — Figure S1. Physiological responses to CO2 exposure in anaesthetized Eln+/+ and Eln+/− mice. Table S1. Brain region volumes (mm3). [file EPH-9999-0-s001.pdf]

Supplemental Figure 1

A

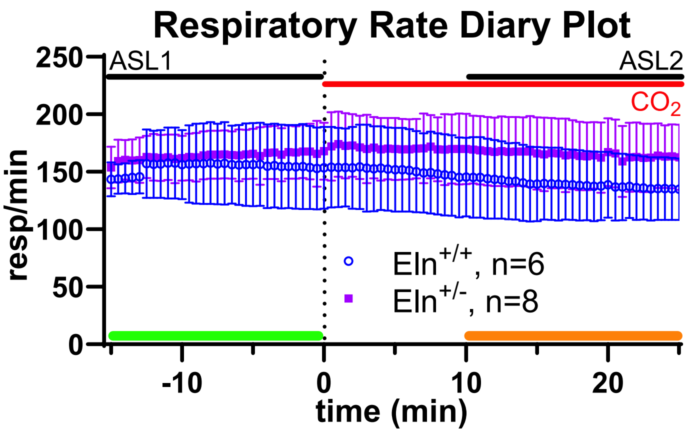

B

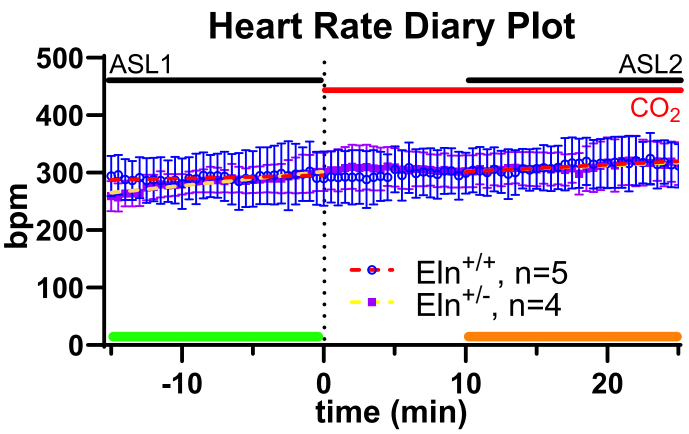

Supplemental Table 1. Brain Region Volumes (mm^3)

	Amygdala		Corpus Callosum		Striatum		Ventricles	
	male	female	male	female	male	female	male	female
**$Eln^{+/+}$**	13.87±0.60	13.75±0.67	15.18±0.69	15.24±0.43	18.98±0.70	19.19±0.62	3.57±0.25	3.25±0.20
**$Eln^{+/-}$**	13.54±0.62	13.37±0.42	15.07±0.64	15.10±0.45	18.82±1.00	18.87±0.76	3.38±0.25	3.36±0.24
